# A Massive Cavernous Mediastinal Haemangioma Causing Superior Vena Cava Obstruction and Extending to the Supraclavicular Space: A Case Report

**DOI:** 10.7759/cureus.104374

**Published:** 2026-02-27

**Authors:** Antonios Charokopos, Andreas Granitsas, Georgios T Stathopoulos, Irene Zarvou, Stella Petrou, Pinelopi Anagnostopoulou, Nikos Chondros, Elena Theofanous, Konstantinos Markakis, Tonia Adamides

**Affiliations:** 1 School of Medicine, University of Cyprus, Nicosia, CYP; 2 Department of Pulmonology, Nicosia General Hospital, Nicosia, CYP; 3 Department of Radiology, Nicosia General Hospital, Nicosia, CYP; 4 Department of Histopathology, Nicosia General Hospital, Nicosia, CYP; 5 Department of Cardiothoracic Surgery, Nicosia General Hospital, Nicosia, CYP

**Keywords:** haemangioma, mediastinal mass, superior vena cava (svc) syndrome, thoracic radiology, vascular tumour

## Abstract

Mediastinal haemangiomas are exceptionally rare, benign vascular tumours and account for a very small proportion of all mediastinal masses. Their symptomatology ranges from an incidental finding to significant mass-mediated compression of vital thoracic structures.

We report a unique case of a massive mediastinal cavernous haemangioma with supraclavicular and axillary extension, which led to central venous obstruction. A 24-year-old man, with a childhood history of a resected supraclavicular cyst, was found to have a symptomatic right-sided heterogeneous mediastinal mass. Computed tomography (CT) angiography identified the hypervascular mass extending from the anterior mediastinum to the supraclavicular fossa, which caused aneurysmal dilatation of the superior vena cava (SVC), with an extensive collateral venous network. Magnetic resonance imaging (MRI) appearance was highly suggestive of a haemangioma, with three interconnecting regions of sizes 7.5 × 6 cm, 9 × 5 cm, and 8.5 × 1.6 cm. The mass was histologically confirmed by transthoracic needle biopsy as a cavernous haemangioma. Due to signs of venous-phase extravasation into the mediastinum and worsening SVC diameter, embolisation was recommended after a comprehensive multidisciplinary meeting.

Our radiopathologic case highlights the diagnostic intricacies of differentiating extremely bulky, but benign, haemangiomas from aggressive mediastinal malignancies. Although surgical excision is the gold standard, the involvement of critical structures, like the SVC, necessitates multidisciplinary discussion and consideration of alternative treatment approaches.

## Introduction

Mediastinal masses are a major cause of clinical concern, even when identified incidentally. When invasion or obstruction of vital structures is involved, significant clinical complications can arise [1]. Among these complications, mediastinal haemorrhage can cause compression of regional vascular or airway structures, with consequent hemodynamic or respiratory symptoms. Superior vena cava (SVC) obstruction, when severe, can lead to facial/trunk swelling and neurological symptoms. Haemangiomas are non-metastasising, usually slow-growing, and sometimes locally compressive benign tumours, which tend to have specific radiologic features on computed tomography (CT) and magnetic resonance imaging (MRI) [2,3].

We present a rare case of a massive mediastinal haemangioma with supraclavicular and axillary extension, causing SVC obstruction in a young man, and discuss the relevant literature.

## Case presentation

A 24-year-old man from Congo, a non-smoker, with a history of a resected right supraclavicular cyst as an infant, visited the Emergency Department of Nicosia General Hospital, Nicosia, Cyprus, after a road traffic accident. Clinical history and laboratory tests showed no signs of infection or hypoxemia. During the workup, he underwent a chest X-ray, which revealed a large space-occupying lesion involving the right upper lung field and right mediastinal border, and a CT chest confirmed the lesion. The medical staff suggested admission for further investigation, but the patient refused due to health insurance reasons. A few weeks later, he visited the Emergency Department again for right-sided chest pain. Pemberton's sign was positive, with mild facial oedema, but he lacked any dyspnoea or neurologic symptoms. A CT-pulmonary angiogram (CT-PA) excluded pulmonary embolism and instead again showed the large mass in the anterior right mediastinum, extending to the right supraclavicular space and lower neck. In addition, it showed progression in the aneurysmal dilatation of the SVC, from 3.5 cm to 3.8 cm, with subsequent compression of the right main branch of the pulmonary artery and the lobar branch for the right lower lobe. Finally, the mass effect caused severe extrinsic narrowing of the right subclavian and internal jugular veins, with a significant collateral venous network, and contralateral dilatation of the left brachiocephalic and subclavian veins. There were internal calcifications, later identified as phleboliths.

**Figure 1:**
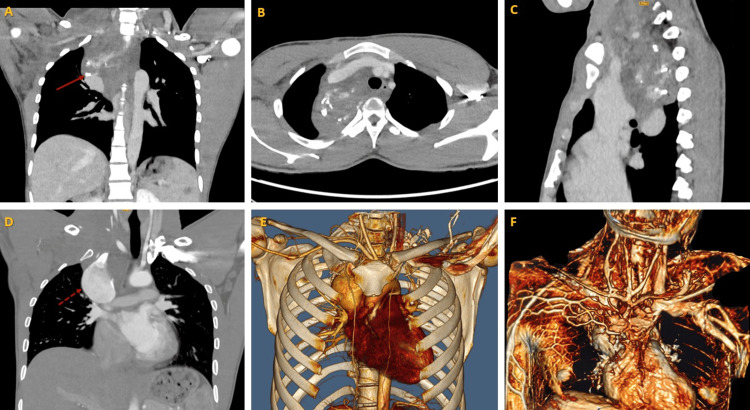
Computed tomography (CT) angiogram of mediastinal mass CT aortic angiogram showing the mediastinal mass that extends above the clavicle in coronal (A), axial (B), and sagittal (C) views; the presence of superior vena cava (SVC) dilation in the coronal CT view (D) and 3D reconstruction view (E); and the presence of superficial dilated chest veins (F). Phleboliths are seen (red solid arrow, A). SVC aneurysmal dilatation is seen (red dotted arrow, D).

A digital subtraction angiography (DSA) was suggested to the patient, but he refused. Instead, an arterial- and venous-phase CT angiography was performed (Figure 1), which revealed, once again, the aneurysm of the SVC, with a diameter of 4.2 cm (increased compared to his previous imaging a few days earlier) and active venous-phase extravasation into the mediastinum. The mass had radiodensity (~60 Hounsfield units) and characteristics compatible with blood, which concerned the radiologist regarding a possible haematoma.

**Figure 2:**
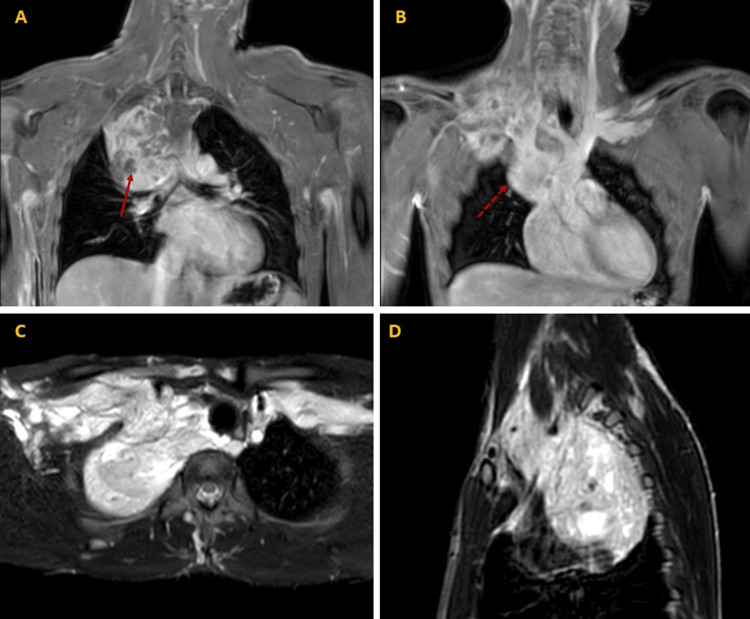
Magnetic resonance angiogram (MRA) of mediastinal mass MRA views of the mediastinal mass, its consequent superior vena cava (SVC) dilatation, and supraclavicular extension in coronal (A, B), axial (C), and sagittal (D) views. Phlebolith is seen (red solid arrow, A). Aneurysmal dilatation of the SVC is seen (red dotted arrow, B). Supraclavicular extension of the mass is particularly evident (C and D).

A subsequent magnetic resonance angiogram (MRA) of the chest showed a heterogeneous mass comprising three connected parts (Figure 2): one adjacent to the right upper lobe (7.5 × 6 cm), with large central vascularity; a second in the right sub- and supra-clavicular area (9 × 5 cm); and a third in the right axillary area and right lateral chest wall (8.5 × 1.6 cm). The lesion appeared to cause dilatation of the SVC and azygos veins, to shift the mediastinum to the left, and to encase the right subclavicular artery and the ipsilateral internal jugular vein. In addition, there was mild uptake of the MRI contrast dye within the mass, and a regional appearance of vascular structures within it. The above findings suggested a haemangioma.

**Figure 3:**
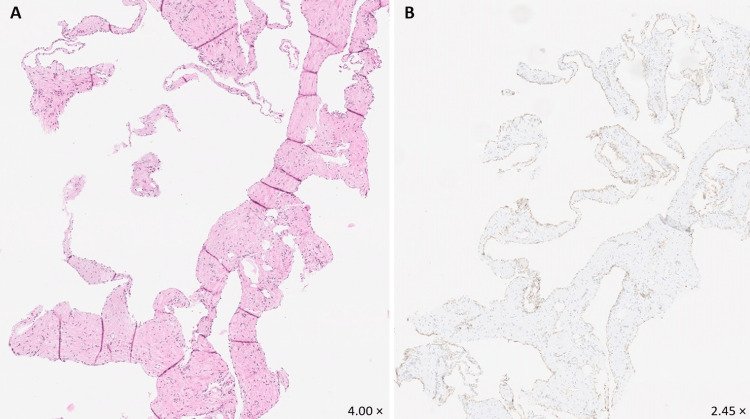
Fine-needle biopsy histological sections, stained with H&E (A) and CD31 (B)

After a multi-disciplinary meeting with thoracic surgeons, vascular surgeons, pulmonologists, and interventional radiologists, a transthoracic fine-needle biopsy (FNB) was planned. The histology specimen revealed tumour-like changes composed of dilated blood vessels, with flattened endothelium and thin fibrous strands, consistent with a haemangioma (Figure 3). Immunohistochemistry was positive for CD31 (+), and negative for D2-40 (-), calretinin (-), CK7 (-), TTF1 (-), CDX2 (-), p63 (-), TdT (-), and PTH (-). The subclassification of the haemangioma was considered to be cavernous. The decision of a second multi-disciplinary meeting favoured embolisation, but the patient refused any further intervention and left the hospital against medical advice.

## Discussion

Mediastinal haemangiomas are benign vascular tumours and only comprise 0.5% of all mediastinal masses. While the classic differential of anterior mediastinal masses includes thymomas, thyroid malignancies, and lymphomas, haemangiomas should also be in the differential diagnostic arsenal of clinicians [1]. The majority occur in the first four decades of life and, while often asymptomatic, they can occasionally compress or invade nearby structures. However, concomitant extrathoracic extension - as in our clinical case - is even rarer and has been described in 2% of mediastinal haemangiomas [4]. Giant haemangiomas are considered those with a diameter greater than 4 cm [2], with a recent radiologic case series reporting sizes between 2 cm and 7 cm [3]. Despite their relatively indolent nature, major complications, including life-threatening haemoptysis and intractable hypoxemia due to associated arteriovenous fistula, have been reported [5,6].

Mediastinal haemangiomas should be distinguished from pulmonary haemangiomas, which are smaller tumours, ranging from 0.3 cm to 2.5 cm within the pulmonary lobes, and which can present as slowly enlarging lung nodules on CT imaging [7,8]. While lobar or sublobar resection is frequently undertaken for pulmonary haemangiomas, mediastinal haemangiomas are intraoperatively found in the anterior (most frequently), middle, or posterior mediastinum, in the thymus gland, and can even require resection of the pericardium [9]. When found in the anterior mediastinum, the main differential diagnoses to consider alongside haemangiomas are thymoma, teratoma, lymphoma, and thyroid malignancy. When found in the posterior mediastinum, the main additional differential entities to consider are neurogenic tumours, such as nerve sheath or ganglionic tumours [10,11]. Castleman disease and hypervascular malignant metastases of extrathoracic tumours should also be considered.

Surgical excision is the treatment of choice, even for giant mediastinal haemangiomas [12]. A case series of 15 patients with mediastinal haemangiomas, with diameters ranging from 3 cm to 16 cm, evaluated the effect of surgical excision extent and subsequent follow-up for recurrence; six patients underwent complete surgical excision, another six underwent subtotal excision, two underwent radiation, and one patient was observed only after biopsy. Major complications included permanent vocal cord paralysis (two patients) and Horner’s syndrome (one patient) [9]. Most showed no progression of disease. The haemangiomas recurred in one patient from the complete excision group, nine years postoperatively, and in another patient from the subtotal excision group, one year postoperatively, requiring re-excision. Mediastinal haemangiomas can progressively enlarge during observation, and, therefore, angiographic gel-foam embolisation of the feeding vessels prior to excision can be a treatment approach for selected cases [13].

Radiological calcification - phleboliths - were seen in our CT images and have been described in 10% to 58% of patients with mediastinal haemangiomas [4]. These phleboliths likely arise from slow blood flow, venous wall damage, and/or organised thrombi in the vascular microstructures of the haemangioma, which can calcify with time [2,14]. A pampiniform (vine-like) growth pattern is also a reliable and sensitive feature of mediastinal haemangiomas, representing their invasive biological behaviour but limited aggressiveness [2]. Both CT and MRI can demonstrate significant heterogeneity within the mass, which is likely due to various internal components (thrombosis, myxoid and fibrous tissue, calcification, fat, and haemorrhage) [2,15]. McAdams et al. identified heterogeneous attenuation at contrast material-enhanced CT and specifically categorised the following four patterns: central (most frequent, 60%), mixed central and peripheral, peripheral, and non-specific increased attenuation [16]. A pattern of delayed, gradually increasing enhancement over time - specifically a "progressive centripetal fill-in" pattern - is highly suspicious for mediastinal haemangioma, and is more frequently identified on contrast MRI rather than CT due to longer scanning times and higher soft-tissue resolution [2]. A summary of cases of mediastinal haemangiomas in adults - including their type, size (all smaller than the current case), presenting symptoms, and treatment strategy - is presented in Table 1.

**Table 1:** Summary of cases of mediastinal haemangiomas in adults, including their type, size (all smaller than the current case), presenting symptoms, and treatment strategy NR, not reported; M, male; F, female; ECMO, extracorporeal membrane oxygenation; NET, neuroendocrine tumour

Authors/year	Patient age (years)/sex	Mediastinal haemangioma		Symptoms/treatment
		Type	Size (cm)	
Petteruti et al.(1993) [11]	57/F	Capillary	4 x 3	Neurologic symptoms/surgical resection
Lee et al. (2006) [13]	23/M	Cavernous	4 x 3 x 1.5	Asymptomatic/angiographic embolisation and surgery
Yamazaki et al. (2006) [17]	61/M	Cavernous	4.2 x 3.2 x 1.7	Asymptomatic/surgical resection
Yoshino et al. (2012) [18]	54/F	Venous	2.7	Asymptomatic/surgical resection
Li et al. (2017) [14]	60/F	Cavernous	NR	Tracheal deviation/surgical resection
Peng et al. (2020) [10]	23/F	NR	8.2 x 4.8	Asymptomatic/surgical resection
Petrie et al. (2020) [12]	85/M	NR	11	Dyspnoea/declined surgery
Huang et al. (2021) [5]	28/F	NR	4 x 5 x 3.5	Haemoptysis/ECMO/surgical resection
Yobita et al. (2021) [19]	73/M	NR	5	Asymptomatic/surgical resection
Mardani et al. (2023) [20]	48/M	Cavernous	12.5 x 10.8 x 6	Cough/surgical resection
Kar et al. (2024) [21]	54/F	Capillary	4.3 x 3.6 x 5	Back pain/NET-like features/surgical resection
Wu et al. (2024) [6]	71/M	NR	5 x 2.5	Pulmonary arteriovenous fistula/surgery

## Conclusions

We report a case of a massive mediastinal haemangioma with supraclavicular and axillary extensions, which caused SVC obstruction. Clinicians should be aware of its unique radiologic characteristics on CT and MRI, including phleboliths, heterogeneous attenuation, and a “progressive centripetal fill-in” pattern. Haemangiomas should be considered in the differential diagnosis of mediastinal masses, especially in the anterior mediastinum. When vital structures are affected, a multidisciplinary diagnostic and treatment approach is beneficial. While surgery is the standard of care in the treatment of mediastinal haemangiomas, angiographic embolisation can be pursued when immediate proximity to major vessels complicates surgical excision.
